# Salvage surgery for pouch-related complication after ileal pouch–anal anastomosis: a report of two cases

**DOI:** 10.1186/s40792-024-01910-0

**Published:** 2024-05-03

**Authors:** Yusuke Izutani, Takayuki Ogino, Yuki Sekido, Mitsunobu Takeda, Tsuyoshi Hata, Atsushi Hamabe, Norikatsu Miyoshi, Mamoru Uemura, Tsunekazu Mizushima, Yuichiro Doki, Hidetoshi Eguchi

**Affiliations:** 1 Department of Gastroenterological Surgery, Graduate School of Medicine, Osaka University, 2-2 Yamadaoka E-2, Suita, Osaka 565-0871 Japan; 2 Department of Gastroenterological Surgery, Osaka Police Hospital, Tennoji-Ku Kitayamacho 10-31, Osaka City, Osaka 543-0035 Japan

**Keywords:** Ulcerative colitis, Ileal pouch–anal anastomosis, Pouch-related complications, Salvage surgery

## Abstract

**Background:**

Pouch-related complications (PRCs), such as pelvic abscesses and perianal complex fistulas, can occur after ileal pouch–anal anastomosis (IPAA) in ulcerative colitis (UC). They are often difficult to treat and require salvage surgery. We report two cases of PRC associated with fistulas.

**Case presentation:**

First case: A 38-year-old man was diagnosed with UC at age 26 years. Four months after the diagnosis of UC, the patient underwent hand-assisted laparoscopic restorative proctocolectomy, IPAA, and ileostomy for acute fulminant UC. Two years after the closure of the ileostomy, the patient developed a perianal abscess and underwent ileostomy reconstruction. He was referred to our department at 35 years of age, because his symptoms did not improve despite repeated seton drainage of a complicated perineal fistula. We diagnosed PRC with a pelvic abscess and complicated pouch fistula and performed salvage surgery. This diagnosis was revised to Crohn’s disease. Second case: A 50-year-old man was diagnosed with UC at age 18 years and was administered high doses of steroids; however, his symptoms did not improve. He underwent restorative proctocolectomy, IPAA, and ileostomy at another hospital. The ileostomy was closed, and his condition stabilized thereafter. At 35 years of age, perianal pain developed, and he was diagnosed with a complicated pouch–perineal fistula. A fistula was observed near the staple line of the ileal end closure on the head side of the pouch. Reconstruction of the ileostomy and seton drainage were performed; however, his symptoms did not improve, and he was referred to our hospital. We diagnosed PRC with a pelvic abscess and a complicated pouch fistula and performed salvage surgery. The resected specimen showed strictures in two locations: at the oral site of the afferent limb (at the pouch) and at the IPAA. Both patients returned to society and are currently outpatients.

**Conclusions:**

We encountered two cases of PRC after IPAA that did not improve with seton drainage or ileostomy. Pouch resection was performed after considering the patient’s quality of life and reintegration into society.

## Background

Standard surgical procedures for ulcerative colitis (UC) and familial adenomatous polyposis include restorative proctocolectomy and ileal pouch–anal anastomosis (IPAA). Pouch-related complications (PRCs) include pelvic abscesses and fistulas near the pouch (pouch–vaginal, pouch–perineal, and pouch–bladder fistulas); they are serious postoperative complications that can lead to sepsis and are one of the most important factors affecting long-term functional outcomes after IPAA [1].

The frequency of PRCs is ~ 2.6–14% [2–4], most of which are IPAA-related fistulas. PRCs often occur within 4–5 months after IPAA, although some cases have occurred as late as 20 months after IPAA [5, 6]. They require multiple restorative procedures and can eventually lead to pouch dysfunction, which has a significant impact on functional outcomes and patient quality of life [7]. Salvage surgery is necessary to improve anal function and quality of life in patients with PRC but is generally difficult in many cases [8]. PRCs often improve with ileostomy; however, pouch resection is required in some cases [9]. Salvage surgery is difficult to perform because of the high degree of adhesion around the pouch and pelvic inflammation. Here, we report two cases of PRC with fistula formation that underwent salvage surgery with favorable outcomes.

## Case presentation

### First case

**Fig. 1:**
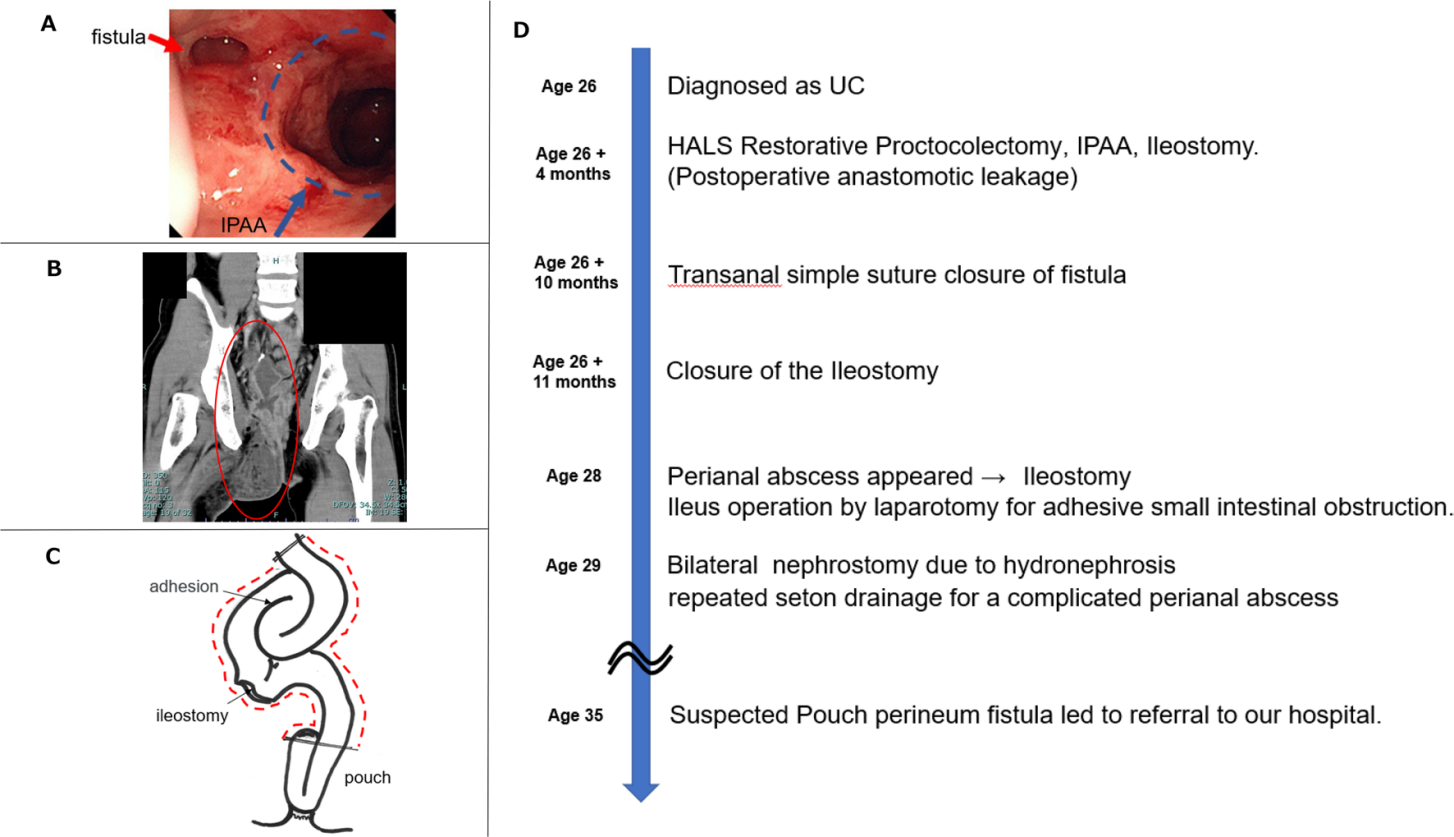
**A** Colonoscopy revealed a fistula near the anastomosis. **B** Computed tomography (CT) showed a perianal abscess. **C** Postoperative small intestinal obstruction developed, and the small intestine, including the ileostomy and part of the cranial pouch, was resected. **D** Timeline from diagnosis of UC to referral to our hospital

A 38-year-old man was diagnosed with UC at the age of 26 years. Four months after the diagnosis of UC, the patient underwent hand-assisted laparoscopic restorative proctocolectomy, IPAA, and ileostomy for acute fulminant UC. Minor leakage at the anastomosis was observed postoperatively but improved with conservative treatment. Although a fistula was observed near the anastomosis, no obvious abscess formation was noted (Fig. 1A). Six months after surgery, simple closure of the fistula was performed using the transanal approach, and the ileostomy was closed 1 month later. Two years after closure of the ileostomy, he developed a perianal abscess (Fig. 1B) and underwent ileostomy reconstruction. However, a postoperative small intestinal obstruction developed, and the small intestine, including the ileostomy and part of the cranial pouch, was resected (Fig. 1C). At age 29, he underwent bilateral nephrostomy for bilateral ureteral strictures caused by chronic pelvic inflammation. Thereafter, his general condition remained stable despite chronic inflammation. He was referred to our department at age 35, because his symptoms did not improve despite repeated seton drainage of a complicated perineal fistula (Fig. 1D).

**Fig. 2:**
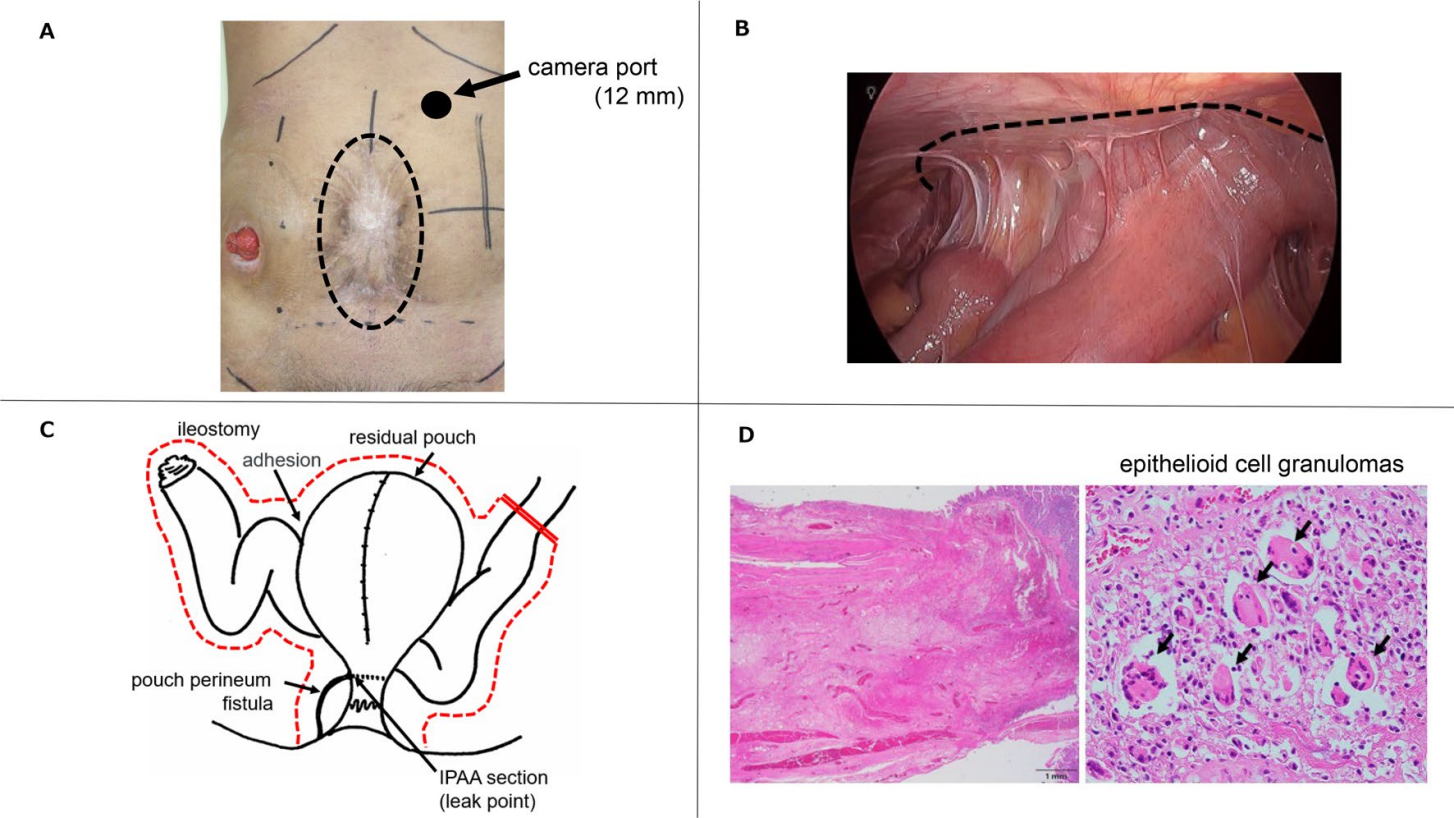
**A** Camera port was inserted into the left upper abdomen, because adhesions were expected in the dotted line area. **B** Intraperitoneal observation revealed small intestine adhesions on the abdominal wall in the dotted line area. **C** Residual pouch containing the complex fistula and the small intestine involved in the pelvic abscess were resected. **D** Histopathological examination showed inflammation in all layers of the pouch, as well as epithelioid cell granulomas

We diagnosed PRC with a pelvic abscess and complicated pouch fistula and decided to perform salvage surgery. We performed residual pouch resection, partial small intestinal resection, and ileostomy reconstruction. The operation time was 686 min, and the blood loss was 800 ml. As the midline wound was expected to have a high degree of adhesion, we utilized laparoscopy for the adhesion dissection and laparotomy for the intestinal manipulation. We inserted the camera port into the left upper abdomen, where we assumed there would be minimal adhesions, and observed the abdominal cavity (Fig. 2A, B). We carefully dissected the adhesions between the small intestine and abdominal wall to expose the residual pouch. Because the small intestine was highly adherent to the pelvis, we used an anal approach.

At this point, we decided to open the abdomen and perform resection of the residual pouch, including the complex fistula, and partial resection of the small intestine involved in the pelvic abscess (Fig. 2C). The midline wound was closed after the scar was trimmed. The residual small intestine was 200 cm in length. Histopathological examination showed inflammation in all layers of the pouch, as well as epithelioid cell granulomas (Fig. 2D), and the diagnosis was revised to Crohn’s disease (CD) postoperatively. Postoperatively, the Alb and CRP values were within normal ranges, although the preoperative Alb and CRP levels were 2.8 g/dl and 10 mg/dl, respectively. His quality of life improved notably, and he returned to society and is now an outpatient.

### Second case

The patient was a 50-year-old man who was diagnosed with UC at 18 years of age and was administered high doses of steroids; however, his symptoms did not improve. He had undergone subtotal colectomy and ileostomy at a previous hospital. Subsequently, the patient underwent residual rectal resection, IPAA, and ileostomy. The ileostomy was closed 8 months after the surgery, and his condition stabilized thereafter. At 35 years of age, perianal pain developed, and the patient was diagnosed with a complicated pouch–perineal fistula.

**Fig. 3:**
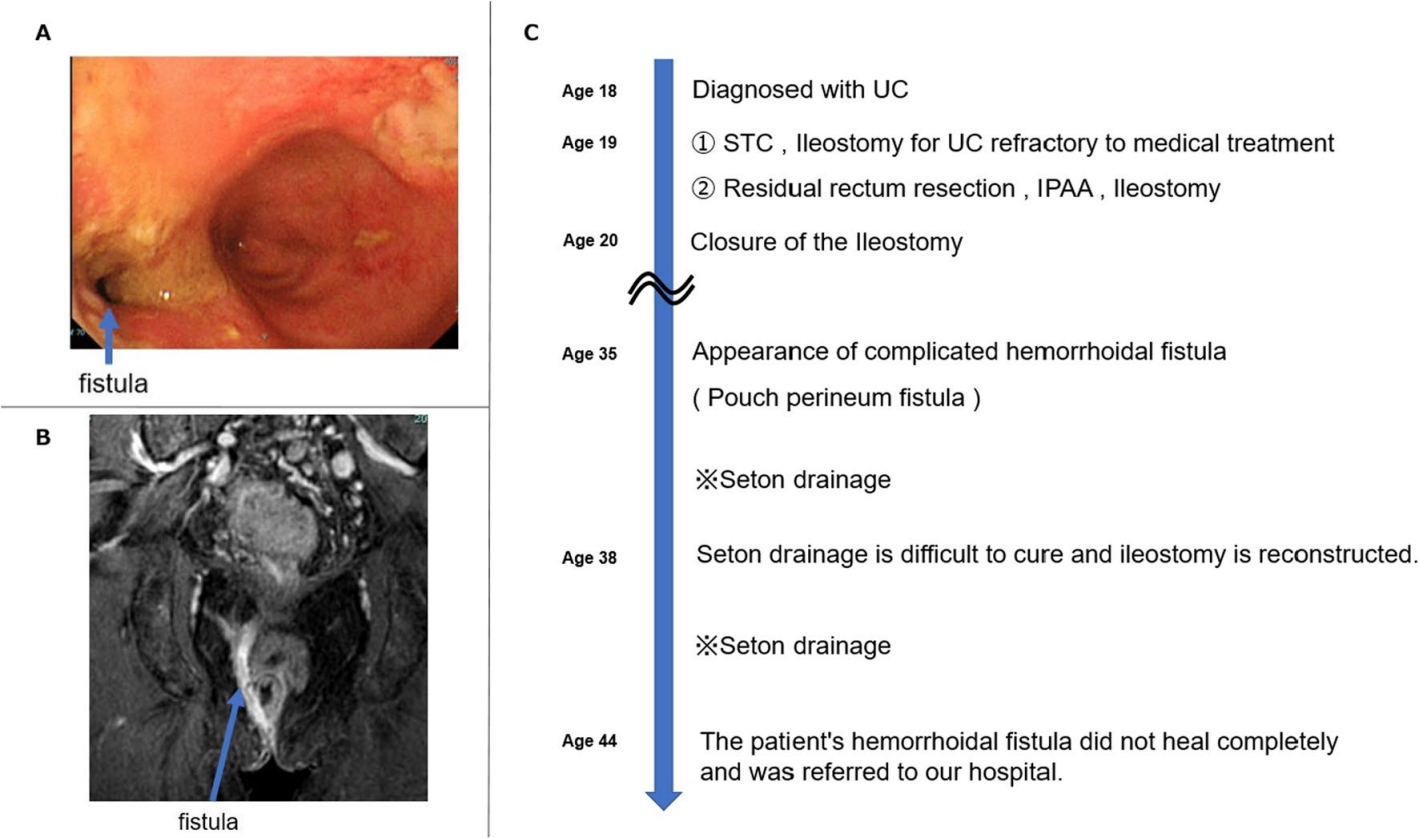
**A**, **B** Colonoscopy and magnetic resonance imaging showed a fistula near the staple line at the ileal end closure on the head side of the pouch. **C** Timeline from diagnosis of UC to referral to our hospital

**Fig. 4:**
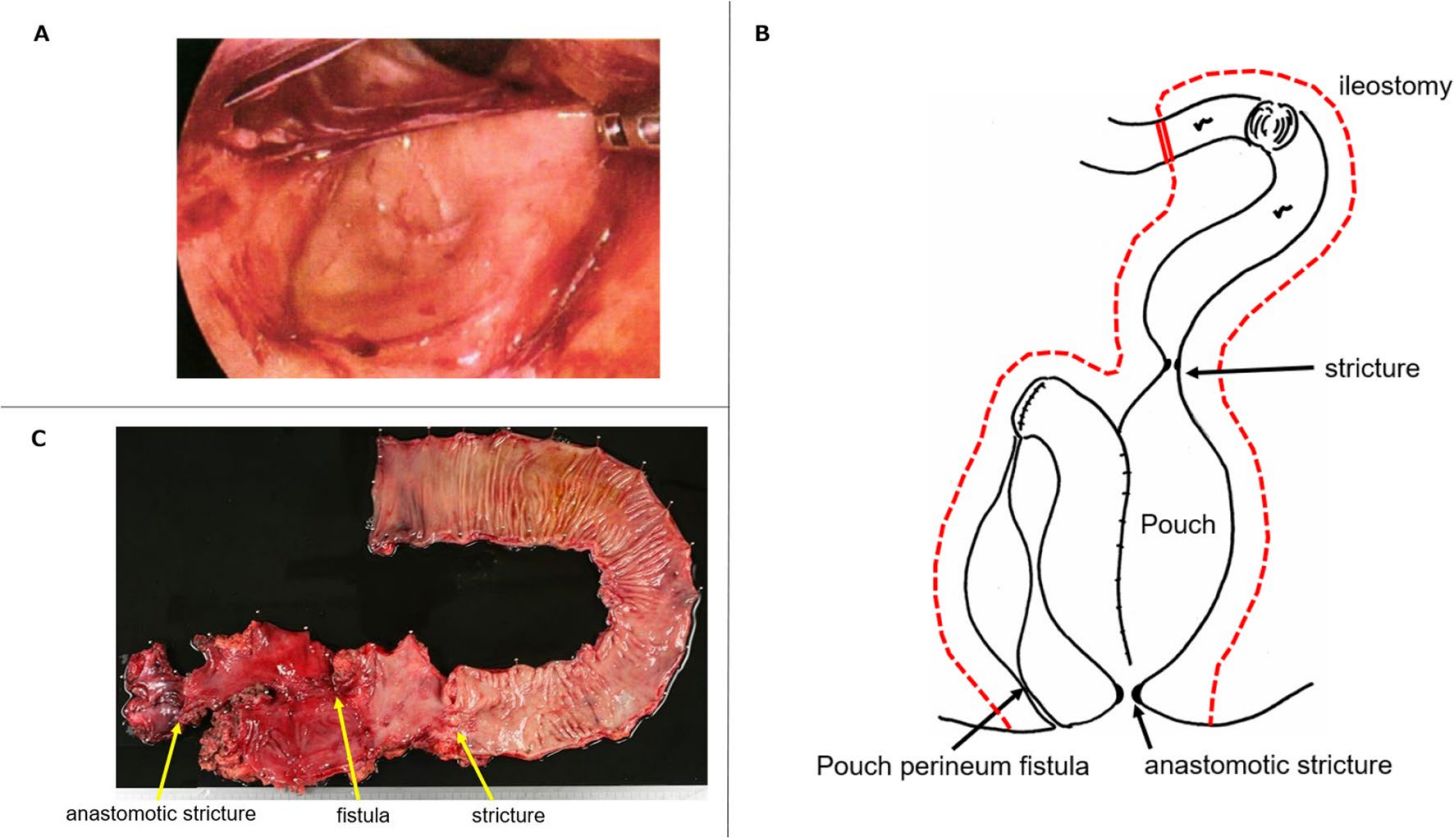
**A** Pouch was highly adherent in the pelvis. **B** Pouch containing the fistula and part of the small intestine, including the ileostomy, were resected. **C** Resection specimen showed stricture in two locations: at the oral site of the afferent limb (at the pouch) and at the IPAA

Colonoscopy and magnetic resonance imaging revealed a fistula near the staple line at the ileal end closure on the head side of the pouch (Fig. 3A, B). Reconstruction of the ileostomy and seton drainage were performed; however, his symptoms did not improve, and he was referred to our hospital (Fig. 3C). We diagnosed PRC with a pelvic abscess and pouch perineal fistula and decided to perform salvage surgery. We performed laparoscopic pouch resection and ileostomy reconstruction. The operative time was 668 min, and the blood loss was 250 ml. The pouch was carefully dissected, because it was highly adherent to the pelvis (Fig. 4A). We performed pouch resection, including fistula resection, using an additional anal approach and partial small intestinal resection, including ileostomy (Fig. 4B). The resected specimen showed strictures in two locations: at the oral site of the afferent limb (at the pouch) and at the IPAA (Fig. 4C). Histopathological examination revealed an inflammatory cell infiltrate mainly composed of lymphocytes in the extensive pouch mucosa but no epithelioid cell granulomas or other findings suggestive of CD. Postoperatively, the CRP level was within the normal range, while the preoperative CRP was ~ 1 mg/dl for a long period of time. In contrast to the preoperative BMI of 17.3kg/m^2^, the patient's nutritional status improved postoperatively. He returned to society and is now an outpatient.

## Discussion

Long-term complications of IPAA include pouchitis and PRC. Pouchitis, defined as chronic active inflammation within the mucosa of the pouch, is a common postoperative complication that occurs in ~ 50% of UC patients with pouches [10, 11]. Pouchitis is often distinguished from PRC, because it often improves with antibiotic therapy and rarely requires surgical treatment [9]. In the present case, both patients developed PRC with pelvic abscess and fistula formation around the pouch.

Risk factors for PRC include early postoperative sepsis, surgical technique (anastomotic tension, poor blood flow to the pouch, etc.), obesity, anastomotic stricture, and diagnostic change to CD after IPAA, which is associated with a higher incidence of PRC than UC [12–17]. In addition, patients with a high cumulative steroid dosage and severe preoperative clinical course are at risk of chronic pouchitis [18, 19]. In Case 1, the diagnosis was changed to CD based on the postoperative pathology. After IPAA, 9% of patients with UC had their diagnosis changed to CD [20]. In Case 2, the patient was treated with high-dose steroids before IPAA. In addition, there were two strictures near the afferent limb at the pouch and anastomosis, and a fistula was observed at the blind end of the pouch (Fig. 4B). Fistulas at the blind end of the pouch are infrequent, with an incidence of < 1% after IPAA [21]. In our case, there was an abscess around the afferent limb of the pouch, and the pathological findings at the site of stricture on the oral side showed inflammatory cell infiltration, capillary hyperplasia, and granulation-like tissue with fibrosis, suggesting that this stricture was caused by chronic inflammation. The stricture on the anal side was caused by anastomotic stenosis. The internal pressure of the pouch may have increased due to strictures at two locations on the oral and anal sides of the pouch, resulting in fistula formation at the blind end of the pouch.

Although PRC can cause pouch dysfunction, it can be treated conservatively if the patients are in good general condition with no evidence of sepsis. However, significant deterioration of the patient's general condition and quality of life is an indication for salvage surgery. PRC often requires salvage surgery such as ileostomy or pouch resection [3–5, 22–26]. In addition, the pouch function rate in patients with CD is 55% at 20 years postoperatively, which is lower than that in patients with UC, and the risk of pouch resection is significantly higher than that in patients with UC [2, 23]. Regarding salvage surgery, it is necessary to select a surgical procedure, such as pouch resection or repeating IPAA, for each patient, taking into account the risk of postoperative complications and the patient's wish. Although the success rate of repeating IPAA increases in patients whose fistula improves after ileostomy [27], neither of our cases showed improvement after ileostomy. In our cases, the indication for surgery was the poor quality of life caused by the fistula with chronic inflammation. The recurrence and severe defecation dysfunction were anticipated if IPAA were reperformed, because the tissue surrounding the previous anastomosis was fibrotic due to pelvic abscess, and sphincter function was impaired due to long-term bowel exclusion of pouch, Therefore, the pouch was finally removed, because the patient’s symptoms did not improve even after seton drainage and reconstruction of the ileostomy.

Regardless of the patient’s symptoms, regular follow-up is required to monitor the pouch status. The patient in Case 1 was not followed up as an outpatient after IPAA. He had a fistula and pelvic abscess that appeared 2 years postoperatively and could have been diagnosed earlier. It is also important to note that small intestinal lesions may appear after restorative proctocolectomy in patients with UC [28]. The patient in Case 2 had a fistula and pelvic abscess 15 years after the IPAA. The cumulative rate of pouch failure after IPAA has been reported to be about 13–15% over a 10-year period [29, 30].

## Conclusion

We experienced two cases of PRC after IPAA that did not improve with seton drainage or ileostomy, and pouch resection was performed considering the patient’s quality of life and reintegration into society. The background and clinical course of PRC are diverse and lead to significant deterioration in quality of life. Treatment of PRC is notably difficult and may require multiple surgeries [31]; in many cases, the surgical difficulty is high due to extensive adhesions. To prevent pouch failure due to PRC, we believe that it is important to establish surgical techniques for pouch creation and to perform periodic pouch scopes to detect PRC in its early stages.

## Data Availability

The data sets supporting the findings and inferences of this case report are included in this article.

## Abbreviations

CD: Crohn’s disease
IPAA: Ileal pouch–anal anastomosis
PRC: Pouch-related complications
UC: Ulcerative colitis
